# Exploring the Bacteriophage Frontier: A Bibliometric Analysis of Clinical Trials Between 1965 and 2024

**DOI:** 10.7759/cureus.56266

**Published:** 2024-03-16

**Authors:** Namrata Dagli, Mainul Haque, Santosh Kumar

**Affiliations:** 1 Karnavati Scientific Research Center (KSRC), Karnavati School of Dentistry, Karnavati University, Gandhinagar, IND; 2 Pharmacology and Therapeutics, National Defence University of Malaysia, Kuala Lumpur, MYS; 3 Periodontology and Implantology, Karnavati School of Dentistry, Karnavati University, Gandhinagar, IND

**Keywords:** visualization, analysis, thematic analysis, network analysis, collaboration analysis, antibiotic resistance, biblioshiny, vosviewer, bibliometric analysis, bacteriophage

## Abstract

In recent years, the rise of antibiotic-resistant bacteria has posed a severe threat to global public health, necessitating innovative and alternative approaches to combat this escalating crisis. Bacteriophages, viruses that infect and replicate within bacteria, have emerged as promising candidates for therapeutic intervention against antibiotic-resistant pathogens. This study delves into the intricate landscape of bacteriophage research, unraveling the trends and impact of research in the field. The analysis considers the chronological evolution of research, identifying key contributors, collaborative networks, and thematic trends that have shaped the trajectory of this rapidly growing field.

Out of 101717 search results in the PubMed database, 163 clinical trials were identified, revealing a dynamic landscape of research activity between 1965 and 2024. The annual scientific publication analysis unveiled fluctuations in the number of publications, indicating an overall increasing trend. Notably, 2011 emerged as a peak year, signifying heightened activity in bacteriophage research. Employing Lotka's law, the authors' productivity analysis illustrated an inherent imbalance in author contributions, with a majority contributing to a single clinical trial. Co-authorship analysis highlighted leading collaborators. Co-occurrence analysis of keywords unveiled thematic clusters, providing insights into the diverse aspects of bacteriophage research. A word cloud emphasized significant terms, while a thematic map categorized themes into various developmental stages. Antimicrobial Agents, Chemotherapy, and Poultry Science were the most relevant journals based on the number of publications. The analysis of countries' contributions revealed the United States as a leading contributor, with Switzerland and China following suit. Collaboration patterns suggested predominantly independent research, with potential for increased international partnerships in certain regions. Additionally, temporal analysis of authors, institutions, sources, and countries revealed productivity patterns, historical context, and research shifts. By scrutinizing a vast array of scientific literature, this investigation aims to provide a panoramic view of how the scientific community has explored the potential of bacteriophages in the context of antibiotic resistance.

## Introduction and background

Bacteriophages represent a fascinating and essential component of the microbial world, serving as natural predators of bacteria. With their unique ability to infect and replicate within bacterial hosts, bacteriophages have garnered significant attention in microbiology and biotechnology. The study of bacteriophages is a rapidly evolving area of research, marked by a wealth of publications exploring various facets of their biology, applications, and interactions with bacterial hosts [1-6].

Their name, derived from the Greek words "bacteria" (meaning bacteria) and "phagein" (meaning to eat), encapsulates their primary role as natural predators of bacteria. Discovered independently by Frederick Twort and Felix d'Herelle in the early 20th century, bacteriophages have since become a focal point of scientific exploration, offering unique insights into the complex dynamics of microbial ecosystems [7-9].

Bacteriophages are remarkably diverse in shape, structure, and genetic composition. From simple polyhedral structures to intricate tails and complex protein coats, these viruses exhibit a wide array of morphologies. Their genetic material can be either DNA or RNA, further contributing to the complexity of bacteriophage taxonomy. The specificity of phages for particular bacterial hosts is a defining characteristic, as they recognize and attach to surface receptors on bacterial cells, initiating the infection process [10].

One of the most intriguing aspects of bacteriophages lies in their pivotal role in shaping bacterial populations and influencing microbial ecosystems. As natural predators, phages play a crucial role in regulating bacterial abundance and diversity, contributing to the balance of microbial communities [11]. Beyond their ecological significance, bacteriophages have emerged as powerful tools in various fields, including biotechnology, food, and medicine [12-14].

In biotechnology, phages are harnessed for their ability to infect bacteria selectively, making them valuable tools for genetic engineering [15,16]. Bacteriophages have been explored for their potential therapeutic applications, particularly in combating antibiotic-resistant bacteria. The concept of phage therapy, which involves using bacteriophages to target and eliminate bacterial infections, has experienced a resurgence of interest as an alternative or complement to traditional antibiotic treatments [17].

Bibliometric analysis, a quantitative method of evaluating and analyzing scientific publications, has become invaluable in mapping the research landscape within a specific field. As we delve into the world of bacteriophages through a bibliometric lens, we embark on a journey to uncover the collaborative networks, seminal works, and research hotspots that define this dynamic and pivotal study area. Through systematic analysis, we aim to provide a nuanced perspective on the growth and impact of bacteriophage research, shedding light on the interconnected web of scientific inquiry that shapes our understanding of these remarkable biological entities.

## Review

Materials and methods

Literature Search Strategy

The bibliometric analysis presented in this study was conducted using a comprehensive search of the PubMed database. The search query comprised the following string- (bacteriophage) OR (phage) OR (bacteriophage research). The date of the online search in PubMed was February 23, 2024, ensuring the inclusion of the most recent publications up to that date.

Inclusion and Exclusion Criteria

Only clinical trials published on bacteriophages were considered for inclusion in the study. All other research papers were excluded, including reviews, editorials, book chapters, and conference papers. 

Data Extraction

Metadata from each selected publication was exported to a text file for the analysis, including title, authors, publication date, journal, abstract, and keywords.

Bibliometric Analysis Tools

Bibliometric analysis was performed using specialized tools, including VOSviewer software (version 1.6.20) and the Biblioshiny app (Rstudio version 4.3.1) [18,19]. VOSviewer (version 1.6.20) was utilized to visualize collaboration co-authorship patterns and keyword co-occurrence. Biblioshiny aided in identifying publication trends over time, collaboration networks, and emerging research trends within the field of bacteriophage research.

Results

A total of 101,717 results appeared in the PubMed database. After applying a filter for article type- clinical trials, only 163 results appeared. The bibliometric analysis identified that these 163 clinical trials on bacteriophage were published in 123 sources by 1254 authors between 1965 and 2024. Additionally, it identified 8.5 co-authors per document, 14.11% international co-authorship, 1194 keywords, and two authors who published single-authored documents.

Annual Scientific Publication analysis

**Figure 1:**
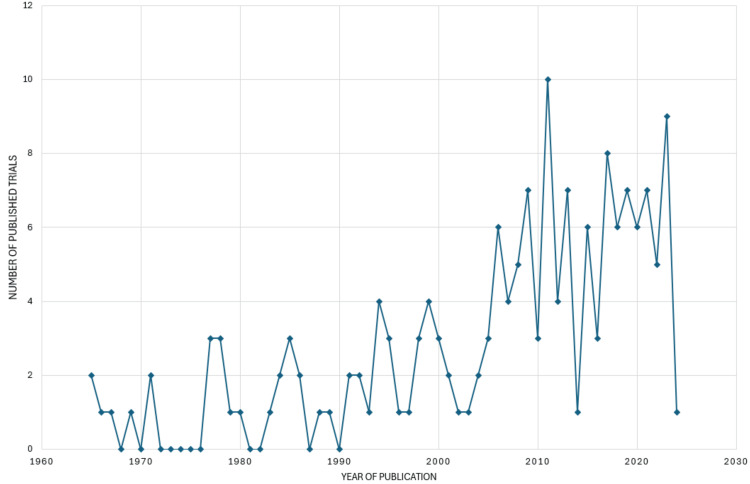
Annual scientific publication of clinical trials. Image Credit: Namrata Dagli

The graph (Figure 1) exhibits a fluctuating pattern, indicating variations in published bacteriophage clinical trials. This fluctuation suggests that the research activity in this domain has not followed a consistent upward or downward trajectory but has experienced changes over time. Despite the fluctuations, the overall trendline suggests a general increase in published papers over the years. This implies a growing interest and emphasis on conducting bacteriophage clinical trials. The upward trajectory of the trendline underscores the rising importance of bacteriophage research, possibly driven by the urgent need to find alternative solutions to combat antibiotic-resistant bacteria. The data indicates that 2011 witnessed the highest number of clinical trials published on bacteriophages, with 10 papers. This peak suggests a particularly active period in bacteriophage research during that year, where researchers and scientists significantly contributed to the literature through clinical trials. Following 2011, the years 2023 and 2017 emerge as notable periods with comparatively more clinical trial publications on bacteriophages. In 2023, there were nine publications, and in 2017, there were eight publications. These years represent other instances of heightened research activity, indicating sustained interest and efforts in exploring the potential of bacteriophages in clinical trials.

Authors’ Productivity Analysis

**Figure 2:**
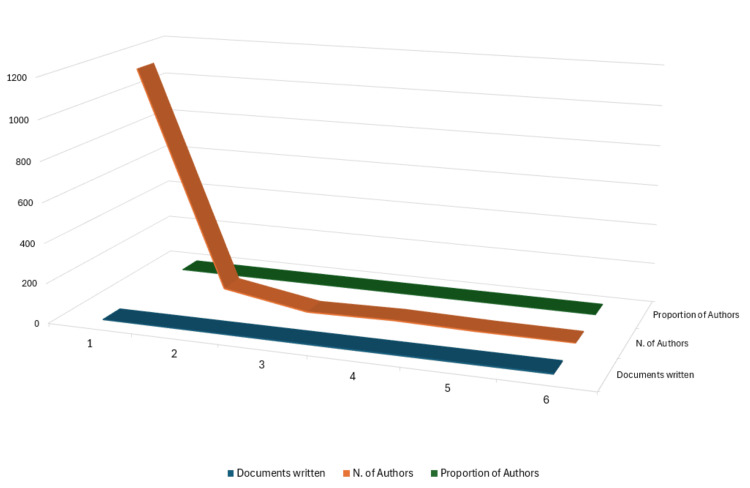
Author’s productivity analysis by Lotka’s Law. Image Credit: Namrata Dagli

Lotka's law, established by Alfred J. Lotka, is a statistical principle that sheds light on the productivity distribution among authors in scientific and academic fields. This law is particularly relevant in bibliometrics and information science, where it helps to understand the patterns of authorship and publication productivity. Lotka's law suggests an inherent imbalance in author productivity, with a small fraction of authors contributing significantly to the overall body of work while most authors contribute less [20]. For this analysis, the Biblioshiny App was used to analyze data, and the results were visualized using Microsoft Excel (Figure 2) to understand how author productivity is distributed in this specific domain. According to the analysis, 1160 authors were identified as having published only one clinical trial on bacteriophages. This aligns with the principles of Lotka's law, indicating that many authors are less prolific, contributing only a single publication to the field.

Analysis of Authors’ Productivity Over Time

**Figure 3:**
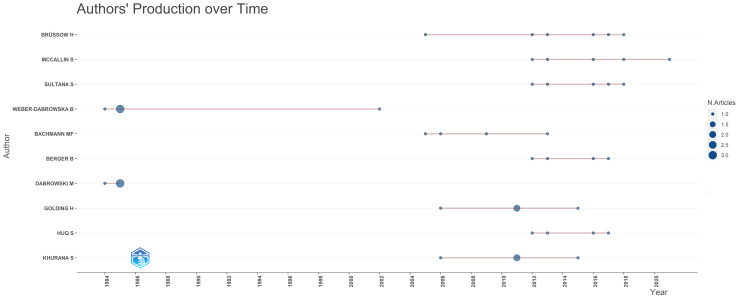
Authors’ productivity over time. N. Articles: Number of Published Clinical Trials. Image Credit: Namrata Dagli

Furthermore, the data revealed that a smaller number of authors, precisely 76, had published 2 clinical trials on the topic. In contrast, only 18 authors published three or more than three clinical trials. This is in line with Lotka's law, where the number of authors with a certain level of productivity is inversely proportional to the square of that number. Figure 3 highlights the authors with the highest number of published clinical trials and their productivity over time.

Co-authorship Analysis of Authors

**Figure 4:**
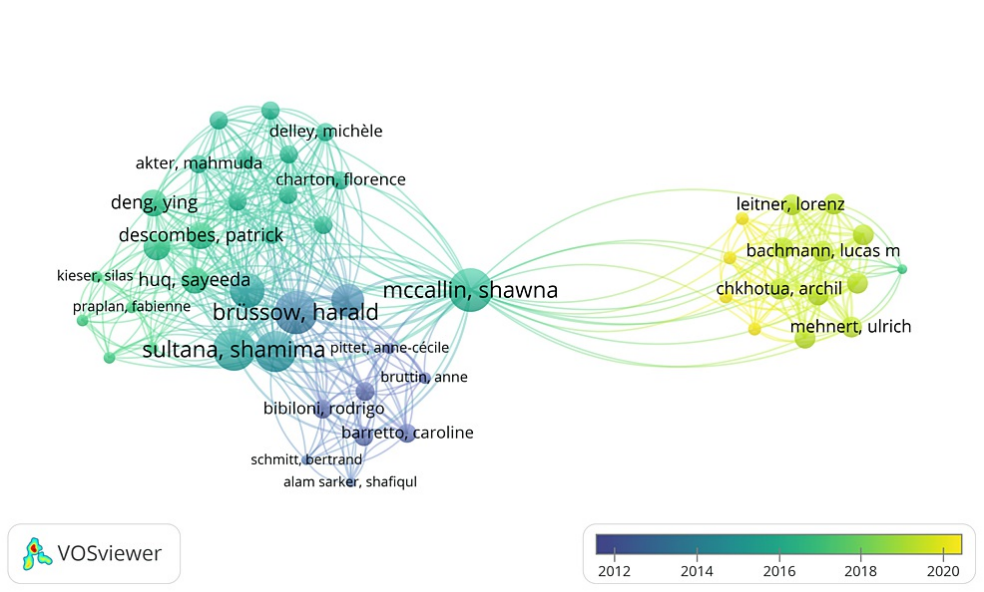
Overlay visualization of co-authorship analysis. Notes:  The size of the circles represents the value of the total link strength of the particular author (Weights: Total link strength, Scores: Average publication year). Image Credit: Namrata Dagli

A total of 1138 authors were identified, and the total strength of their co-authorship links was calculated using VOSviewer. The authors with the highest total link strength were selected for the analysis. The most extensive connected set, which comprised 49 items, was included in the overlay visualization (Figure 4). In this network, four clusters were identified, connected by 445 links, totaling 573 link strengths. Overlay visualizations prove valuable for comprehending the temporal distribution of authors' contributions, aiding in identifying trends or patterns in their research output over time. They offer insights into the timeline of specific authors' or groups of authors' research contributions and help grasp the evolving nature of collaborations. In the co-authorship analysis, authors serve as nodes in a network, connecting lines as links, and the total link strength represents the cumulative measure of collaboration strength between two authors. The thickness of the links between authors reflects this total link strength, with thicker links indicating stronger collaborations. Notably, the analysis reveals that Shawna McCallin boasts the highest total link strength (57), closely trailed by Harald Brussow (56) and Shamima Sultana (55). Their respective numbers of co-authored publications are 5, 6, and 5.

Co-authorship Analysis of the Institution

**Figure 5:**
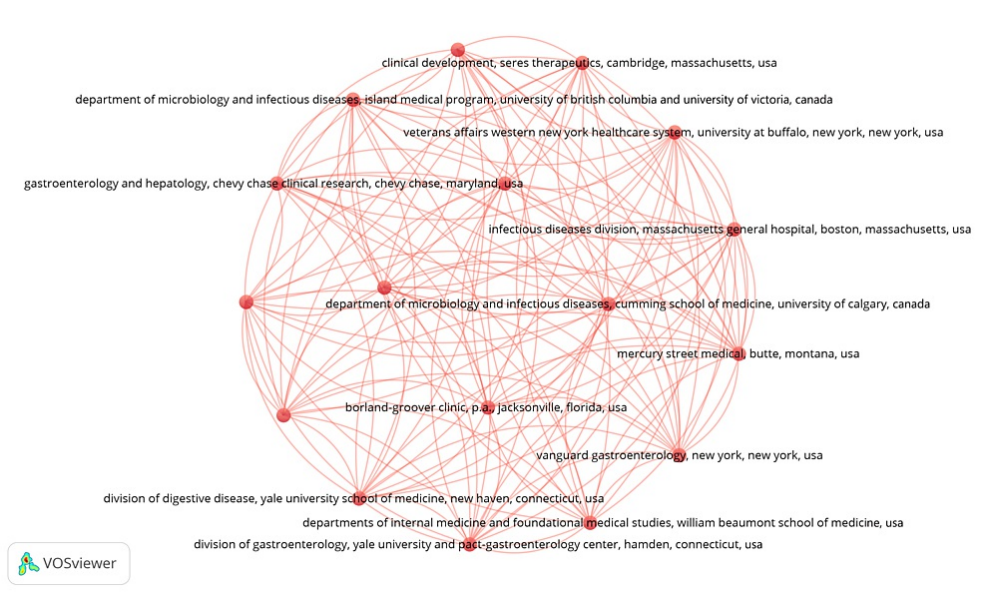
Network visualization of co-authorship analysis of institutions. The size of the circles represents the number of links between them (weights: Links). Image Credit: Namrata Dagli.

A total of 310 organizations were identified, each with at least one published research paper. The total strength for co-authorship links was calculated for each organization using VOSviewer, and those organizations with the greatest total link strengths were selected for the co-authorship analysis. The most extensive set of connected items, comprising 17 items, was included in the network visualization, forming a single cluster with 136 links (Figure 5). In the co-authorship network depicted in Figure 5, nodes represent institutions, and links represent collaborations between these institutions. The weight of each "link" provides information about the degree of cooperation between two institutions, quantifying and visualizing the strength of relationships in the co-authorship network. The size of each circle is determined by the number of links or co-authorship relationships associated with that institution. Larger circles indicate institutions with more collaborations or links in the co-authorship network. The analysis underscores that each of the 17 institutions depicted in Figure 4 has one publication and 16 links. Two of these 17 institutions are in Canada, while the remaining 15 are in the USA.

Institutions’ Production Over Time

**Figure 6:**
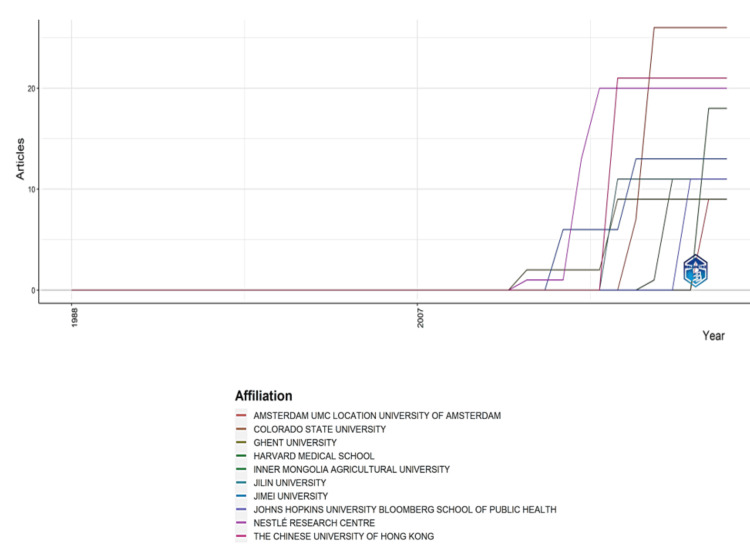
Institution’s production over time. UMC: University Medical Center. Image credit: Namrata Dagli

The graph (Figure 6) shows the most contributing institutions regarding the number of published clinical trials and their productivity trends over time. Amsterdam University Medical Center emerges as the top contributor, having published the highest number of clinical trials, closely followed by Colorado State University, Ghent University, and Harvard Medical School. The institutions mentioned have published over 15 clinical trials, indicating a sustained and substantial contribution to the research landscape. The graph allows these institutions to observe productivity trends over time. It visually represents how their research output has evolved, offering insights into increased or decreased activity periods and their impact on the broader scientific community.

Co-occurrence Analysis of the Keywords

**Figure 7:**
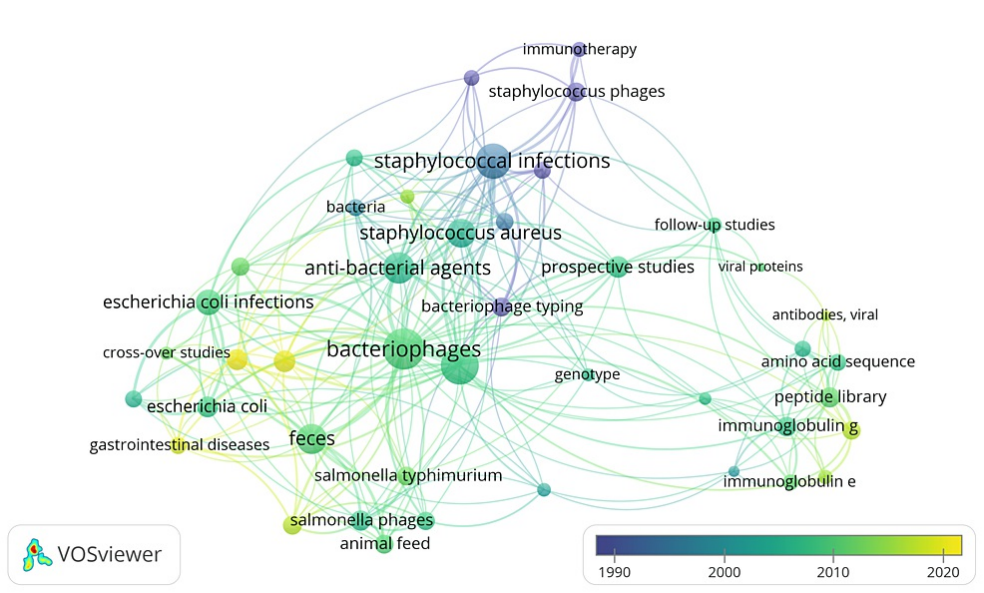
Overlay visualization of the cooccurrence analysis of keywords (weights: Total link strength, Scores: Average publication year). The size of the circle represents the value of total link strength. Image Credit: Namrata Dagli

A total of 863 keywords were identified. When the threshold was set to 5, only 76 keywords appeared. The total strength was calculated for each of them, and the keywords with the highest total link strength were included in the overlay visualization generation. After removing nonspecific and nonrelevant keywords from the analysis, four clusters of 43 items, linked with 245 links and 514 total link strengths, are depicted in Figure 7. The overlay visualization depicts keyword co-occurrence patterns, with the size and color of the connections between keywords representing the total link strength. Additionally, it provides information about the average publication year for each keyword, which helps to understand the thematic evolution of research topics related to bacteriophage. 

The items included in the four clusters are mentioned in Table 1. The keywords in cluster 1 indicate that most clinical trials related to the bacteriophage are follow-up studies and prospective studies involving peptides or proteins on the surface of bacteriophages to study interactions with antibodies, identify epitopes, and analyze molecular sequences. Also, it indicates that the research covers various aspects such as allergen studies, amino acid sequence analysis, antibody formation, genotype analysis, and peptide libraries. The keywords in cluster 2 collectively suggest a focus on researching antibacterial agents, bacterial infections, and the use of bacteriophages, particularly *Staphylococcus* phages, as potential tools or therapies in combating bacterial infections, especially in the context of drug resistance. They also indicate clinical trials on specific bacterial strains, such as *Pseudomonas* and *Staphylococcus*, using bacteriophages in phage therapy. The keywords in cluster 3 collectively indicate research on phage therapy for *Escherichia coli* (*E. coli)* infections and gastrointestinal diseases. Phage therapy involves using bacteriophages, specifically coliphages targeting *E. coli*, as a biological therapy to combat bacterial infections.

**Table 1:** Items in the various clusters identified in the co-occurrence analysis of the keywords.

Cluster 1 (14 items)	Cluster 2 (12 items)	Cluster 3 (10 items)	Cluster 4 (7 items)
allergens	antibacterial agents	bacteriophages	animal feed
amino acid sequence	bacteria	biological therapy	antibody, bacterial
antibodies, viral	bacterial infection	coliphages	Feces
antibody formation	bacteriophage typing	cross-over studies	Probiotics
epitopes	drug resistance, bacterial	double-anonymized method	Salmonella Enteritidis
follow-up studies	immunotherapy	Escherichia Colli	Salmonella phages
genotype	microbial sensitivity tests	Escherichia Colli infections	Salmonella Typhimurium
immunoglobulin E	mupirocin	gastrointestinal diseases	
immunoglobulin G	Pseudomonas infections	gastrointestinal microbiome	
molecular sequence data	Staphylococcal infections	phage therapy	
peptide library	Staphylococcus Aureus		
prospective studies	Staphylococcus phages		
sensitivity and specificity			
viral proteins			

Additionally, the keywords cross-over studies and double-anonymized method suggest the experimental design of the clinical trials on bacteriophage. The focus is likely on investigating the impact of phage therapy on the gastrointestinal microbiome. The keywords in cluster 4 collectively suggest research on probiotics, particularly bacteriophages targeting Salmonella strains, in animal feed. This research likely focuses on mitigating the risk of bacterial contamination, specifically Salmonella Enteritidis and Salmonella Typhimurium, by applying antibodies and phages. Fecal analysis might be involved in assessing the effectiveness of probiotics in reducing bacterial presence in the digestive system of animals. 

Word Cloud

**Figure 8:**
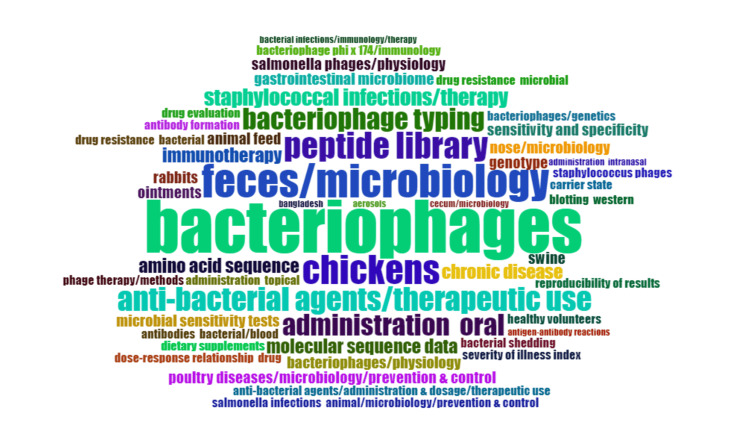
Word cloud showing the most frequently used keywords. The size of the fonts represents the frequency of occurrence of these words in research papers on bacteriophage. Image Credit: Namrata Dagli

A word cloud is a visual representation of text data where words are displayed in varying sizes and colors. The size of each word in the cloud is typically proportional to its frequency or importance in the given text. The more frequently a word appears in the text, the larger and bolder it occurs in the word cloud. The word cloud in Figure 8 is generated using the Biblioshiny App. It visually summarizes the most significant terms in bacteriophage-related published literature, making it easy to identify patterns, themes, or keywords. The most frequent words are human, female, male, adult, infant, and aged. We removed all these nonspecific ages- and gender-related words to identify those more relevant to the topic. The most common words suggest that most animal research has been done on chickens, followed by swine and rabbits. The other most frequently used words are amino acid sequencing, molecular sequencing, Staphylococcal and Salmonella infection, physiology of bacteriophages, antibacterial agents, drug-resistant bacteria, peptide library, bacteriophage typing, animal feed, and microbiological analysis of animal feces.

Thematic Map

**Figure 9:**
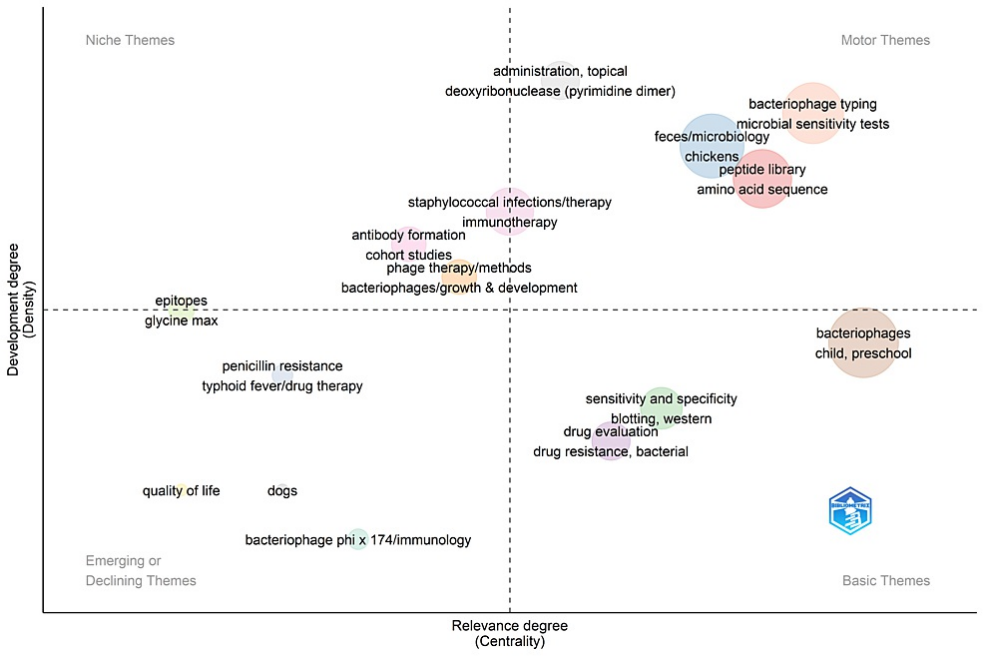
Thematic map categorizing the themes related to bacteriophage research based on their development and relevance. Image credit: Namrata Dagli

The thematic map (Figure 9) generated using the Biblioshiny app (Rstudio version 4.3.1) categorizes the themes into four categories: Motor, Basic, Niche, and Emerging or Declining terms. The motor themes are well-developed and relevant, including bacteriophage typing, microbial sensitivity tests, the microbiology of feces, chickens, peptide library, amino acid sequence, topical administration, and pyrimidine dimer- deoxyribonuclease. The Neche themes are well-developed and isolated, including antibody formation, growth, and development of bacteriophages, phage therapy, and epitopes. The primary themes relevant to the topic, which are not well developed, include sensitivity and specificity, western blotting, bacteriophages, preschool children, drug evaluation, and bacterial drug resistance. The emerging or declining themes, including quality of life, bacteriophage phi, immunology, penicillin resistance, dogs, and drug therapy for typhoid fever, are poorly established. The most pertinent themes related to bacteriophage are bacteriophage typing and microbial sensitivity tests. The thematic map highlights well-established and emerging focus areas in the field of study.

Most Relevant Sources and Their Production Over Time

**Figure 10:**
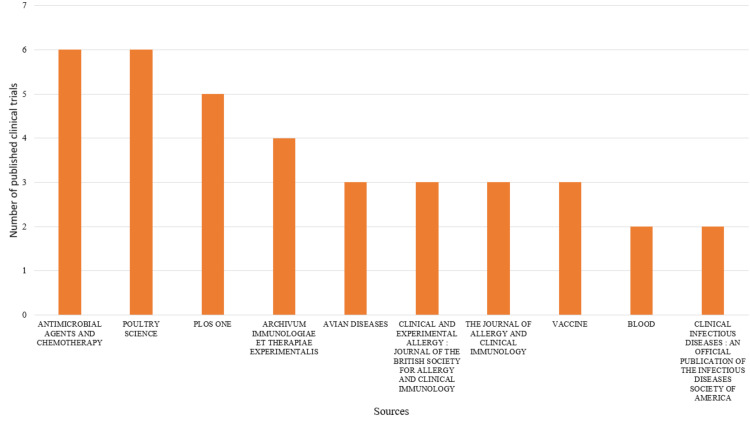
The most relevant sources based on the number of published clinical trials. Image Credit: Namrata Dagli

**Figure 11:**
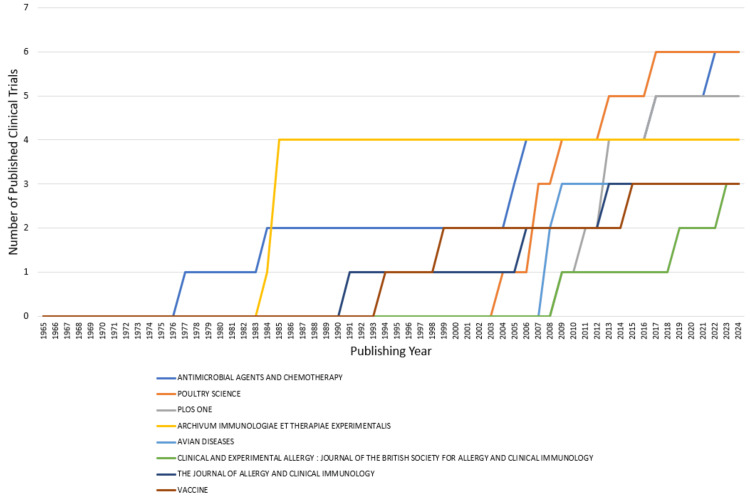
Source’s production over time. Image Credit: Namrata Dagli

Figure 10 depicts the most relevant journals based on the number of published clinical trials on bacteriophage. The journals with the highest number of publications are Antimicrobial Agents and Chemotherapy and Poultry Science, followed closely by Plos One and Archivum Immunologiae Et Therapiae Experimentalis. Figure 11 shows the publications by these most contributing journals over time. Peaks and troughs in the graph might correspond to key historical or scientific events. Identifying these periods helps researchers understand the context and factors influencing the production of sources. 

Most Relevant Countries, Their Production Over Time, and the Collaboration Between Them

**Figure 12:**
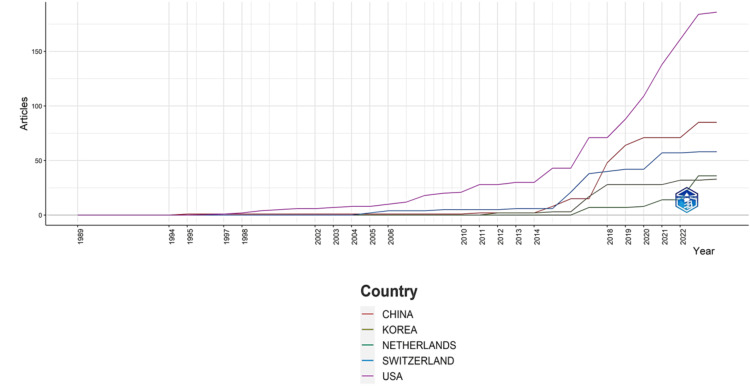
Most countries are based on the number of published clinical trials on bacteriophage and their production over time. Image Credit: Namrata Dagli

Figure 12 illustrates the temporal evolution of published clinical trials on bacteriophage by the most contributing countries based on the number of publications. Notably, there is a discernible surge in publications post-2014, indicating a growing interest in bacteriophage-related clinical trials. The United States consistently emerges as the foremost contributor, leading in the number of clinical trials since 1997. Switzerland held the second position in productivity from 2004 to 2017, after which China assumed the role. This dynamic shift reflects changes in global research contributions over time.

**Figure 13:**
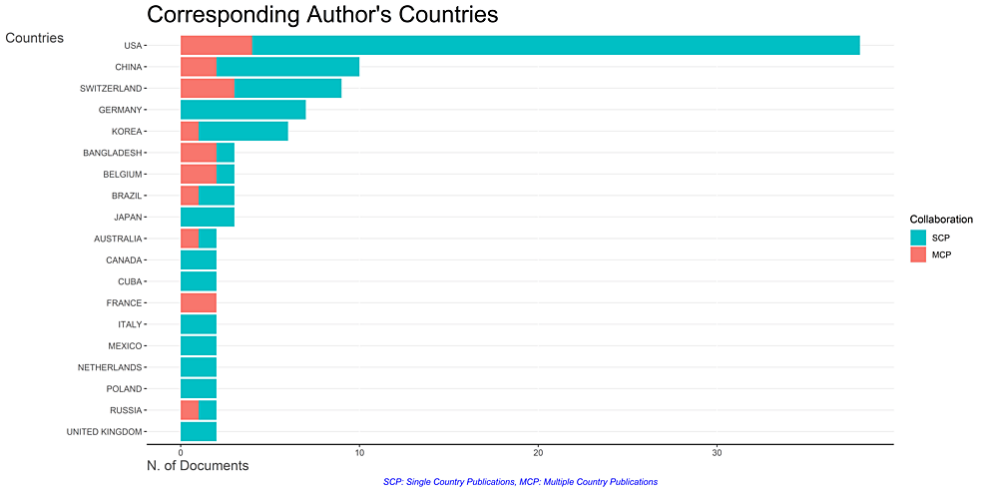
Collaboration frequency of the countries with the highest number of published clinical trials on bacteriophage. Image Credit: Namrata Dagli

Figure 13 provides insights into collaboration frequencies among countries conducting clinical trials on bacteriophage. Single-country publications predominate, suggesting a significant proportion of research is conducted independently. The United States exhibits the highest collaboration frequency, closely followed by Switzerland. Intriguingly, Germany, Japan, Canada, Cuba, Italy, Mexico, Netherlands, Poland, and the United Kingdom have not collaborated with other nations. This points to potential opportunities for fostering international research partnerships in these regions.

**Figure 14:**
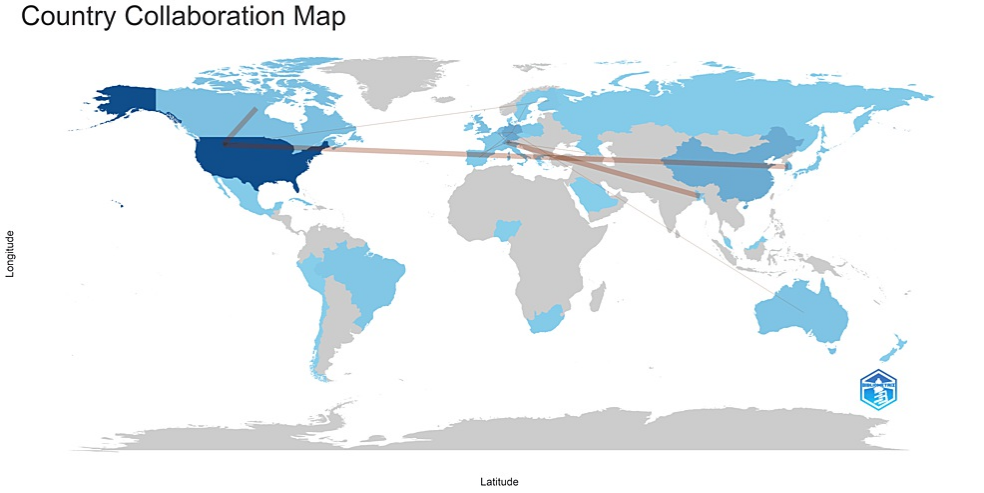
Country collaboration map. Notes: The intensity of blue color represents the number of published clinical trials on bacteriophage. The connecting lines represent the collaboration between the countries. Image Credit: Namrata Dagli

In Figure 14, the country collaboration map visually represents collaborative networks in bacteriophage-related clinical trials. The map suggests sparse collaborations globally, emphasizing the specialized and relatively independent nature of research in this field. The color intensity on the map reflects the number of publications, with darker shades indicating higher publication numbers. Furthermore, the thickness of connecting lines between countries signifies the strength or volume of collaboration, with thicker lines indicating more substantial collaborative efforts. Together, these figures provide a comprehensive overview of the global landscape of bacteriophage-related clinical trials, highlighting trends, collaborative dynamics, and areas for potential research cooperation.

Discussion

The PubMed database yielded 101,717 results, which, after filtering for clinical trials, resulted in 163 relevant studies on bacteriophages published across 123 sources by 1,254 authors between 1965 and 2024. The analysis indicates an average of 8.5 co-authors per document, with 14.11% international co-authorship and 1,194 keywords identified. The publishing trend analysis highlights the dynamic nature of clinical trial publications on bacteriophages, with fluctuations in annual numbers but an overall increasing trend. Notably, 2011 saw the highest number of publications, with subsequent peaks in 2023 and 2017 underscoring periods of intensified research and publication activity in the bacteriophage clinical trials, possibly driven by the urgent need to combat antibiotic-resistant bacteria. The authors' productivity analysis, aligned with Lotka's law [20], reveals that most authors contribute only to a single publication, while a smaller group accounts for multiple publications. This uneven distribution in author productivity aligns with the principle that a small fraction of authors contribute significantly to the literature. This insight provides a nuanced understanding of the collaborative landscape and emphasizes recognizing and fostering contributions from prolific and less prolific authors.

Co-authorship analysis highlights the collaboration between the authors and institutions with the most published clinical trials on bacteriophage. Shawna McCallin, Harald Brussow, and Shamima Sultana emerged as critical collaborators, while institution productivity shows Amsterdam University Medical Center as the leading contributor. Analyzing the productivity trends of these institutions over time provides a deeper understanding of their impact on the research landscape.

Keyword co-occurrence analysis reveals thematic clusters that comprehensively view the research focus areas. The keywords in cluster 1 indicate immunological studies on bacteriophages, which focus on understanding the interactions between bacteriophages and the immune system. The analysis of cluster 2 reveals that most of the bacteriophage research as an antibacterial agent in case of antibiotic-resistant bacterial infections has been done on Pseudomonas, Staphylococcus Aureus, and Staphylococcus phages. The research involved Staphylococcal phages predominantly. Analysis of cluster 3 indicates bacteriophages, particularly coliphages, have been used in phage therapy for gastrointestinal Escherichia coli infections. It also suggests a link between phage therapy and gastrointestinal microbiome. The analysis of cluster 4 indicates that the research on Salmonella Enteritidis, Salmonella Typhimurium, and Salmonella phages is mainly done on animals. Overall, the analysis provides an overview of the different research areas related to bacteriophages, highlighting their potential applications in various fields, including immunology and antibacterial therapy. We found many research papers related to the topics supporting the findings of our study [21-26].

The thematic map categorizes themes into motor, fundamental, niche, and emerging or declining categories, elucidating well-established and emerging research areas. The identified categories in the thematic map provide insights into the distribution of research emphasis within the field, offering a nuanced view of established and evolving themes. This information can guide researchers about potential areas for future exploration or concentration. The motor themes are well-developed themes that are relevant to the study. The keywords identified as motor themes imply a research focus on bacteriophage interactions with bacteria, emphasizing bacteriophage typing and microbial sensitivity testing. Bacteriophage typing involves the classification of these viruses, which is crucial for understanding their diversity. The microbial sensitivity tests likely explore the effectiveness of bacteriophages against specific bacterial strains, offering potential therapeutic insights. Including "feces, chicken" suggests a particular interest in bacteriophages in chicken feces, hinting at research into their role in the chicken gut microbiota or potential applications in poultry health. Using a peptide library and amino acid sequencing indicates a molecular-level investigation, likely aimed at identifying specific peptides involved in bacteriophage-bacteria interactions. Overall, the keywords collectively point to a comprehensive exploration of bacteriophage research, encompassing classification, sensitivity testing, molecular analyses, and microbiological analysis of chicken feces. The primary themes are the themes that are relevant to the topic but not well-developed. These basic themes indicate that the therapeutic use of bacteriophage in bacterial drug resistance warrants more attention. The niche and emerging themes are not particularly relevant to the study; instead, they offer information about themes that are co-studied alongside the main topic.

The most relevant journals and countries contributing to bacteriophage research are identified. Relevant journals included Antimicrobial Agents, Chemotherapy, and Poultry Science. The United States is leading in publications, followed by Switzerland and China. Collaboration analysis underscores independent and collaborative research efforts globally, suggesting opportunities for enhanced international cooperation. Overall, the findings underscore the evolving landscape of bacteriophage research and its growing significance in combating antibiotic resistance and addressing various infectious diseases.

We found a very few bibliometric studies on similar topics. A bibliometric analysis of phage therapy in the Web of Science database by Maimaiti et al. concluded that the United States, China, and the United Kingdom were the most productive countries. The Polish Academy of Sciences was the most contributive institution. Frontiers in Microbiology and Applied and Environmental Microbiology were the most productive and co-cited journals. Regarding keywords, research focuses include phage biology, phage against clinically essential pathogens, phage lysis proteins, phage therapy, biofilm-related research, and recent clinical applications [27]. Another bibliometric analysis and review of randomized controlled trials on bacteriophage revealed an upward trend in citations, indicating that phage therapy has garnered significant attention. The study identified the treatment indications for bacteriophage therapy are bacterial diarrhea, urinary tract infections, infected burn wounds, chronic otitis, chronic venous leg ulcers, and chronic rhinosinusitis. Three studies reported a statistically significant outcome difference when comparing single-phage therapy with standard care or placebo. The study recommended meticulously addressing critical elements, including the quality of phage preparation, sensitivity testing, titer, and dosages, and access to the infection site and stability, in future trials to effectively translate research findings into clinical practice [28]. Another bibliometric analysis identified was related to the antitumor effect of bacteriophages [29]. The study concluded that Ganoderma lucidum, Poria, Morus alba, and Cordyceps sinensis are the most popular antitumor bacteriophages. The high-frequency keywords identified were "Ganoderma lucidum triterpenoid," "Ganoderma lucidum acid," and "Biological activity." The results are not directly comparable as these studies focus on one aspect of bacteriophage research or different databases and articles published during various periods.

Future studies in bacteriophage research should focus on understanding the factors behind the temporal variations in clinical trial publications, especially during peak years like 2011, 2023, and 2017. Further exploration is needed into leading contributors, their collaborative patterns, and their impact on the research field. A deep dive into well-developed and emerging themes will provide insights into the evolving research landscape. Further exploration is needed to understand the potential application of phage therapy across various fields by investigating the relationship with the most frequently used keywords. Additionally, analyzing the surge in publications in the most contributing countries post-2014 and the changing dynamics of their contributions will contribute to a comprehensive understanding of bacteriophage research. Opportunities for fostering international collaborations should be explored, particularly in regions with limited collaborative efforts, to enhance the global landscape of bacteriophage-related clinical trials.

Bibliometric analysis, while a valuable tool for assessing trends and patterns in scientific literature, has inherent limitations that should be considered. Firstly, it is acknowledged that the analysis may not capture all relevant publications due to potential variations in database coverage and search query specificity. Databases may vary in their scope and coverage of journals, leading to the exclusion of certain publications pertinent to the research question. Additionally, the precision of the search queries used in the bibliometric analysis can impact the inclusivity of the retrieved publications. Secondly, the interpretation of results is limited to the availability and accuracy of metadata provided in the selected publications. Incomplete or inaccurate metadata, such as author affiliations, keywords, or citation data, can compromise the reliability of the analysis, affecting the validity of conclusions drawn from the bibliometric data.

## Conclusions

The bibliometric analysis revealed a fluctuating pattern in annual scientific publications, suggesting variations in research activity. Despite fluctuations, an increasing trend indicates growing interest and emphasis on bacteriophage clinical trials. Authors' productivity analysis aligned with Lotka's law, highlighting that many authors contribute only to one publication. Co-authorship analysis identified prominent authors. Amsterdam University Medical Center has been recognized as the most contributing institution. Co-occurrence analysis of keywords provides an overview of the different research areas related to bacteriophages, particularly coliphages, Staphylococcus phages, and Salmonella phages, highlighting their potential applications in various fields, including immunology and antibacterial therapy, the most frequently used research themes. Relevant journals, countries, and their collaboration patterns were also explored. The United States was identified as a leader in publications and international collaboration. Furthermore, examining the temporal aspects of authors, institutions, sources, and countries unveiled patterns in productivity and provided insights into historical context and shifts in research focus. Overall, this analysis provides valuable insights into the landscape of bacteriophage clinical trials, emphasizing emerging trends and opportunities for research collaboration.
